# Transient Light Emitting Devices Based on Soluble Polymer Composites

**DOI:** 10.1038/s41598-018-24816-y

**Published:** 2018-04-23

**Authors:** Yingying Chen, Hang Lu, Fei Xiu, Tao Sun, Yamei Ding, Juqing Liu, Wei Huang

**Affiliations:** 1 Key Laboratory of Flexible Electronics (KLOFE) & Institute of Advanced Materials (IAM), Jiangsu National Synergetic Innovation Center for Advanced Materials (SICAM), Nanjing Tech University (NanjingTech), 30 South Puzhu Road, Nanjing, 211816 China; 2 Key Laboratory for Organic Electronics and Information Displays & Institute of Advanced Materials (IAM), SICAM, Nanjing University of Posts & Telecommunications, 9 Wenyuan Road, Nanjing, 210023 China

## Abstract

Building transient electronics are promising and emerging strategy to alleviate the pollution issues from electronic waste (e-waste). Although a variety of transient devices comprising organic and inorganic materials have been described, transient light emitting devices are still elusive but highly desirable because of the huge amount of lighting and display related consumer electronics. Here we report a simple and efficient fabrication of large-area flexible transient alternating current electroluminescent (ACEL) device through a full-solution processing method. Using transparent flexible AgNW-polymer as both electrodes, the devices exhibit high flexibility and both ends side light emission, with the features of controlled size and patterned structure. By modulating the mass ratio of blue and yellow phosphors, the emission color is changed from white to blue. Impressively, the fabricated ACEL device can be thoroughly dissolved in water within 30 min. Our strategy combining such advances in transient light emitting devices with simple structure, widely used materials, full solution process, and high solubility will demonstrate great potential in resolving the e-waste from abandoned light-emitting products and expand the opportunities for air-stable flexible light emitting devices.

## Introduction

In the past decade, transient electronics have been widely explored largely because electronic waste (e-waste) has been a growing serious global problem due to the rapid development of consumer electronics, such as displays, lighting, computers, mobile phones and other entertainment electronics^1–5^. Currently, e-waste is mainly disposed by several approaches including landfill, burning, as well as acid leaching, all of which pose severe risks to environment and human health^6^. In this regard, harmless electronic products that can degrade or dissolve naturally into environment without e-waste are highly desirable, developing transient electronics would make a great contribution to alleviating the global pollution from e-waste.

Transient electronics (also named soluble electronics), with a key feature that they can afterward physically disappear and degrade to the surrounding environment with no or minimum impact^7^, has made great progress. Normally, they are fabricated based on soluble electrodes, soluble active materials and soluble substrates. For example, several conductive materials, including zinc, magnesium, iron, silver nanowires (Ag NWs) and carbon nanotubes have been served as soluble electrodes^8,9^. Meanwhile, a variety of natural and synthetic polymers, such as silk^10–12^, Zinc oxide^13^, small organic molecular species^14^, polyvinyl alcohol (PVA)^15^ and polyvinyl pyrrolidon (PVP)^16^ have been used as soluble active materials and substrate compounds to construct transient electronic devices, such as thin film transistors^17^, memory devices^18^, sensors^19^ and other transient electronics^20^. However, studies on transient light emitting devices which account for a large part in the lighting and display market are still absent, the quantity of light emitting devices related products is about 42 million tonnes per year globally^21^. Therefore, it is urgent to develop facile method, especially full-solution process, for the large-area and scalable fabrication of transient light emitting devices.

Herein, for the first time, we report a full solution processing method for the large-area and scalable fabrication of transient ACEL devices. AgNW-PVA and AgNW-PVP composite films are used as bottom and top soluble electrodes, respectively. The soluble active layer is made of inorganic phosphor (ZnS:Cu and ZnS:Cu,Mn)-PVP composites. The fabricated device shows high flexibility and both ends side emission, with different size and shape patterns. By modulating the mass ratio of the two phosphors in active medium layer, white and blue light emission can be realized. Impressively, the devices can totally disintegrate in water. Moreover, the transient ACEL devices are fabricated through a full solution process, paving the way for low-cost and large-area manufacturing of light-emitting products.

## Results

**Figure 1:**
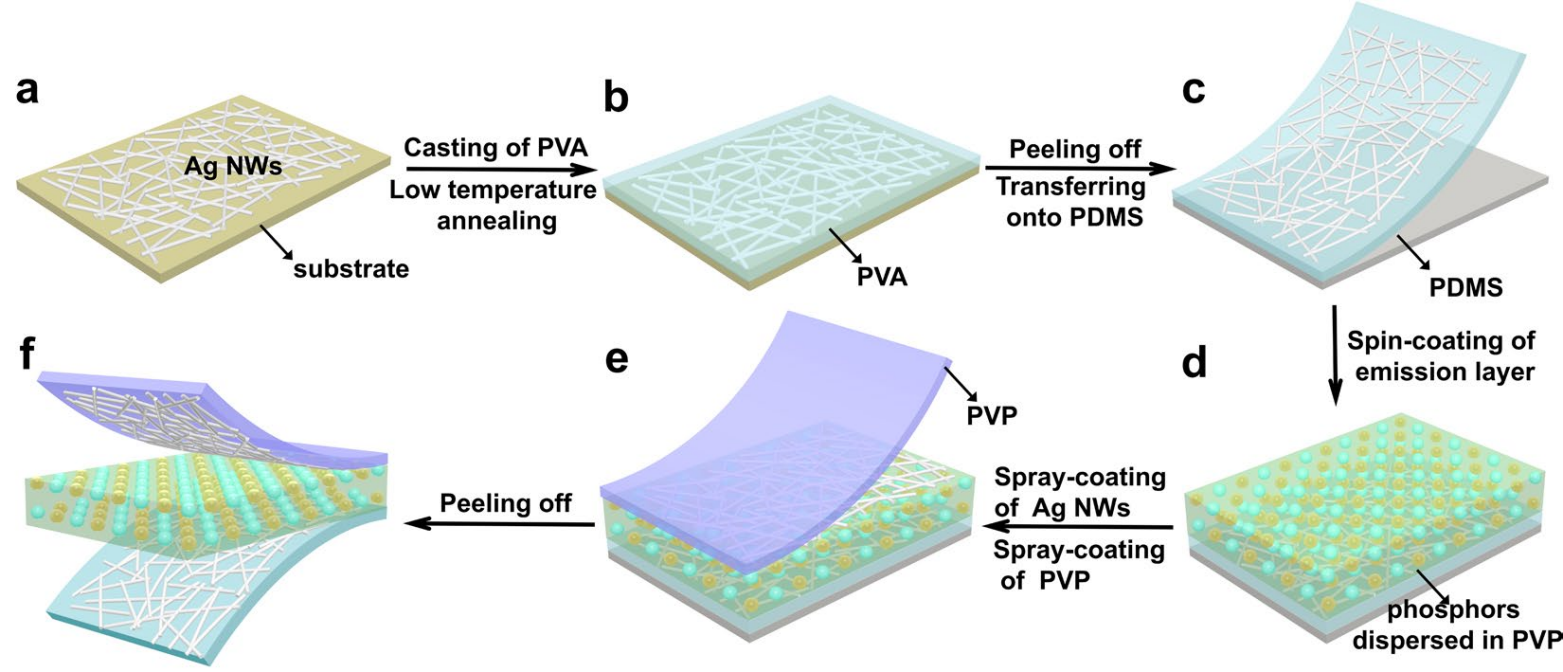
Schematic illustration of the fabrication process of the transient and flexible ACEL device. (**a**) Spray-coating of Ag NW thin film on plastic substrate. (**b**) PVA coating onto the surface of Ag NW film. (**c**) Peeling off and transferring of AgNW-PVA electrode onto PDMS substrate. (**d**) Spin-coating of the phosphors-PVP composite layer upon the electrode surface. (**e**) Spray-coating of Ag NWs and PVP sequentially as the top electrode. (**f**) The schematic of the sandwich structure device.

Figure 1 schematically illustrates the fabrication process and typical configuration of transient ACEL device, where AgNW-PVA film, phosphors (mixture of ZnS:Cu and ZnS:Cu,Mn)-PVP composite, and AgNW-PVP film are used as the bottom electrode, emissive layer, and top electrode, respectively. The fundamental light-emitting mechanism of phosphor-based ACEL device is known to be solid-state cathode luminescence that the light emission arises from impact excitation of the emitting layer by hot electrons accelerated through an inorganic layer^22^. Firstly, Ag NWs are spray-coated on a pre-cleaned plastic substrate followed by a casting of PVA aqueous solution to prepare AgNW-PVA bottom electrode. Subsequently, ZnS:Cu and ZnS:Cu,Mn microparticles mixed in PVP ethanol solution are spun onto the bottom electrode as active layer. Water solvent for PVA and ethanol for PVP are used to avoid compromising the integrity of underlying layers while deposing over layers, thus high quality multi-layers are achieved^23^. Finally, Ag NWs and PVP solution is spray-coated separately on active layer surface as top electrode. This full-solution process without utilizing thermal evaporation paves a way for the low cost and large-area fabrication of electronic devices.

**Figure 2:**
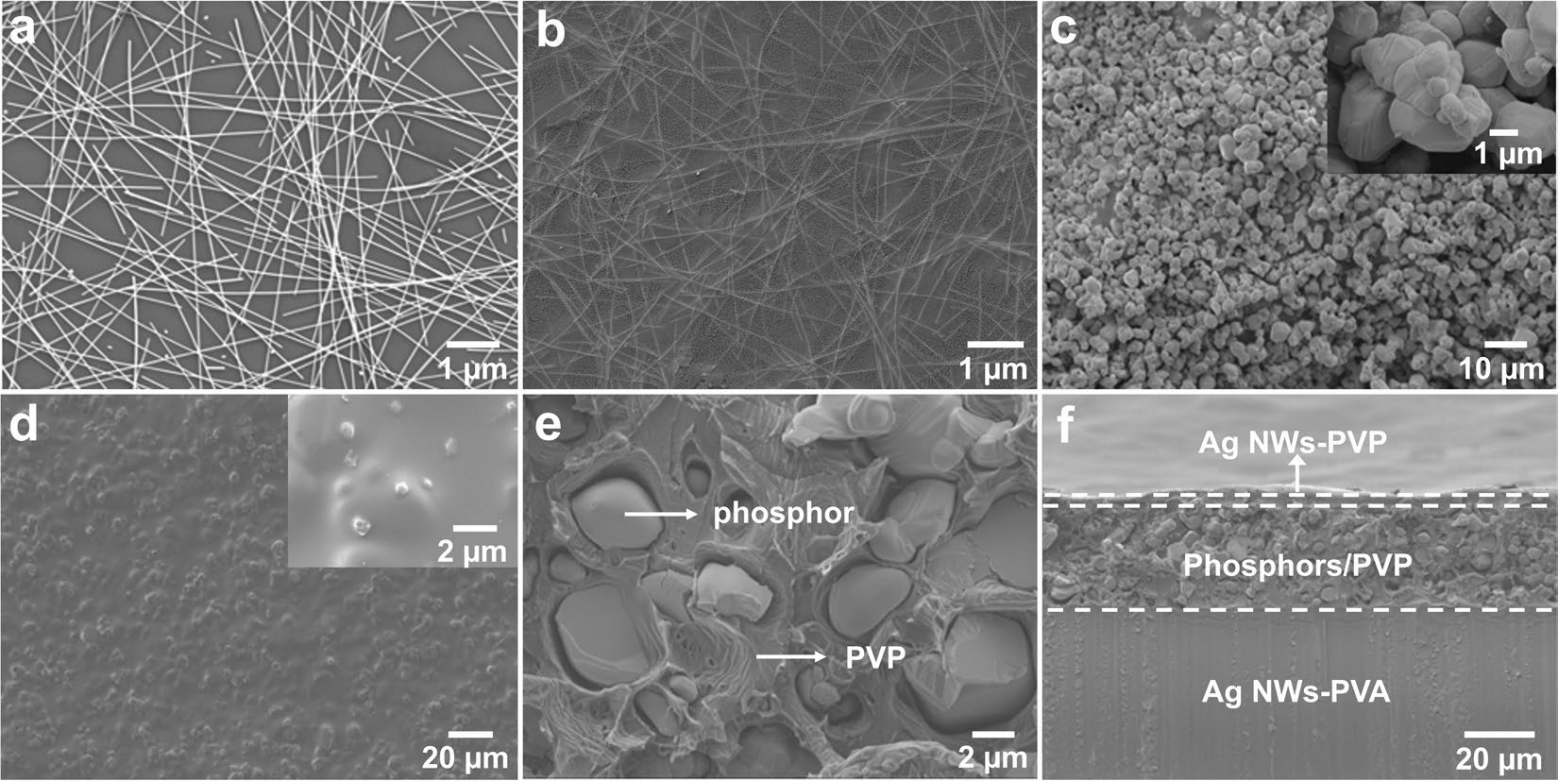
SEM characterization of the fabricated device. (**a**) SEM image of Ag NW networks; (**b**) SEM image of AgNW-PVA electrode; (**c**) Morphology of ZnS:Cu,Mn/ZnS:Cu microparticles; (**d**) Morphology of the mixture phosphors-PVP composite film; (**e**) Enlarged view of the cross-section of phosphor microparticles embedded in PVP; (**f**) Cross-section observation of the device geometry.

The morphology of Ag NW networks, AgNW-PVP electrode and phosphors-containing active layer, as well as the device geometry is examined with scanning electron microscopy (SEM). Figure 2a shows that Ag NWs have a high aspect ratio with an average diameter of 30 nm and an average length of 11 µm. The surface morphology of AgNW-PVA electrode reveals that Ag NWs are embedded and anchored in PVA substrate firmly (Fig. 2b). The strong adhesion of Ag NWs with PVA not only guarantees the stability of electrodes, but also improves their mechanical flexibility without sacrificing the conductivity, which is further confirmed by repeated bending tests (Supplementary Fig. S1). The mixed phosphors exhibit irregular ball shapes with an average diameter of 4 μm (Fig. 2c), they are uniformly dispersed in PVP active medium layer (Fig. 2d,e). The participation of PVP not only endows the active layer a high dielectric permittivity for alter current light emission, but also acts as binder to tightly bind the phosphor particles for film-forming ability. Obviously, cross-section image of the fabricated device demonstrates that a typical sandwich structure with an active layer thickness of 35 μm is observed in Fig. 2f.

**Figure 3:**
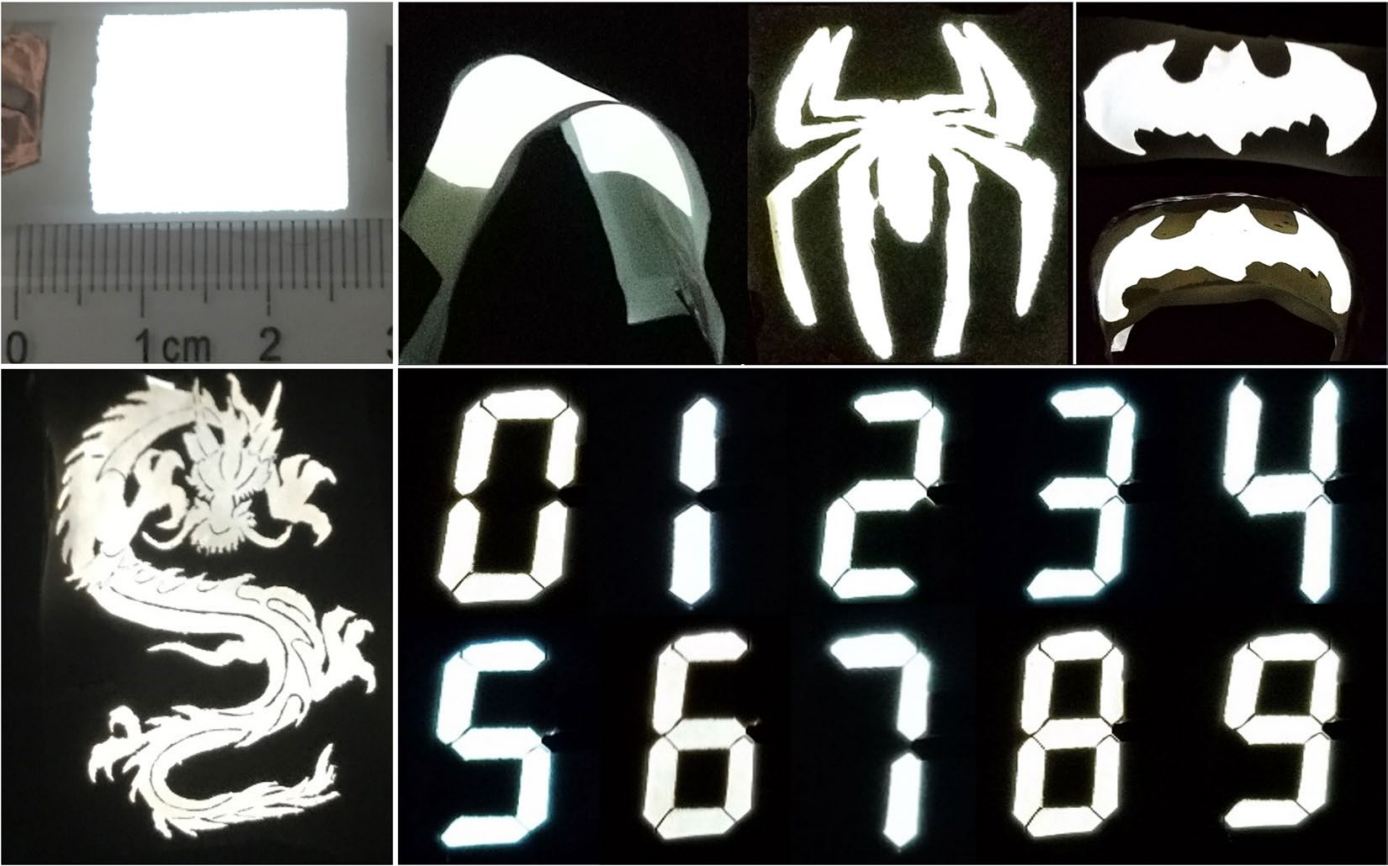
Photographs of the large-area flexible device. The flexible device under flat and mechanical bending state with different size and shape patterns.

Basing on a full solution process method, large-area and flexible light emitting devices have been fabricated and exhibit white light emission under flat and bent state (Fig. 3). It is worth pointing out that the fabricated device can be patterned into different shapes with a precise control, such as a spider, a bat, the Chinese dragon, as well as digital. Particularly, light emission could be extracted from both ends side of device with top emission and bottom emission simultaneously because both AgNW-polymer electrodes are transparent. Moreover, by modulating the mass ratio of the two phosphors, pure blue and patterned ACEL devices with high flexibility could also be obtained (Supplementary Fig. S2). All these advances make the flexible ACEL devices feasible for architectural lighting or as portable devices.

**Figure 4:**
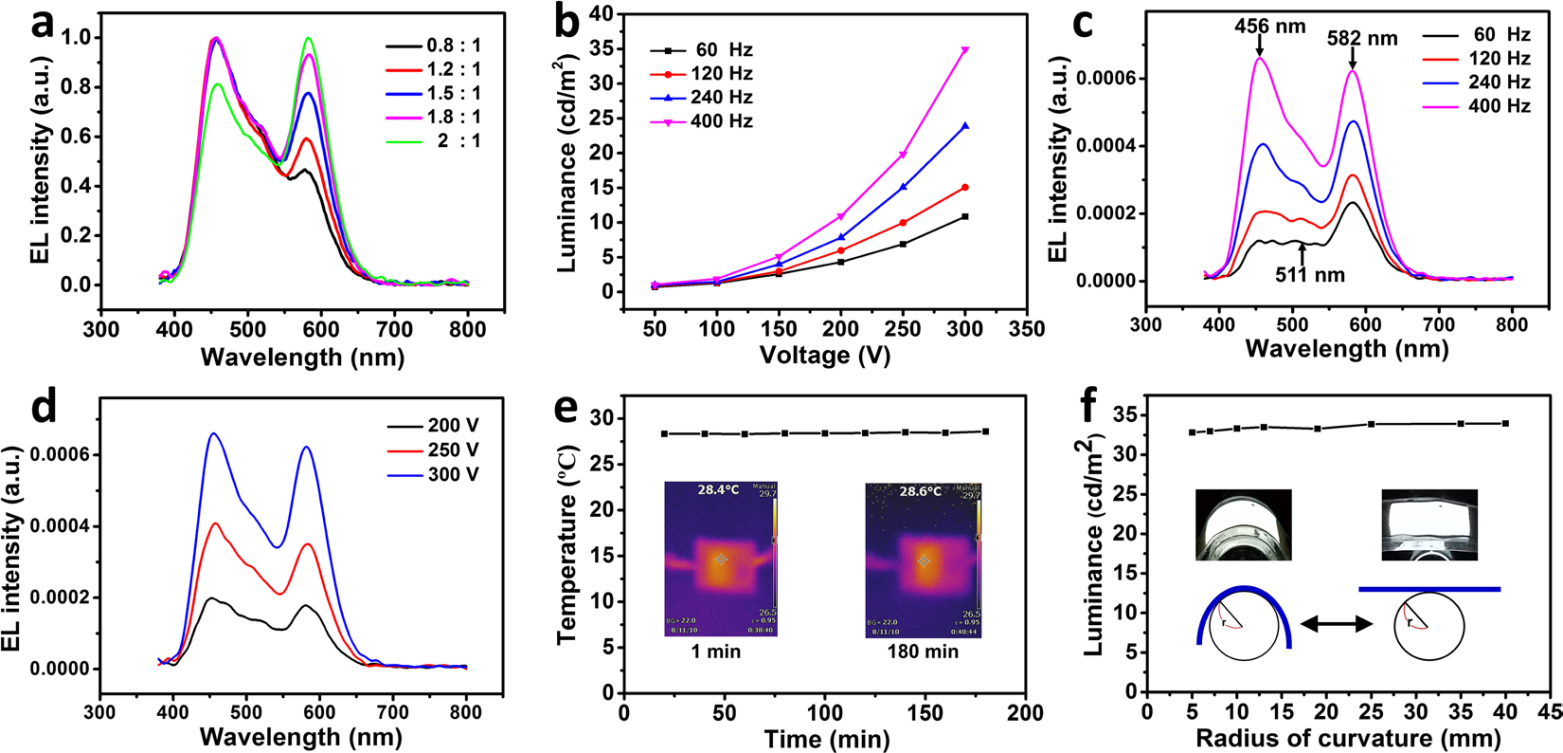
Optoelectronic characterization of white light emission devices. (**a**) The normalized electroluminescence (EL) spectra of the device with different proportions of phosphors. (**b**) Luminance versus alternating voltage properties of the device under different frequencies. Relative EL emission spectra at different (**c**) frequencies and (**d**) voltages. (**e**) The temperature change profile of the device under operation, inset shows the image of infrared temperature. (**f**) Photograph and schematic illustration of white light emitting device as a function of bending radius.

Optoelectronic characteristics of white ACEL devices are investigated systematically. Figure 4a shows the normalized EL spectra of five device samples with different mass ratios of ZnS:Cu, Mn and ZnS:Cu phosphors. All spectra consist of two major peaks centered at 456 nm from ZnS:Cu and 582 nm from ZnS:Cu,Mn. Moreover, the emission color are strongly dependent on the proportion of the two phosphors. Noted that increasing ratio of ZnS:Cu,Mn and ZnS:Cu phosphor is accompanied with the decrease of relative emission intensity from ZnS:Cu. Meanwhile, as expected for the CIE color coordinators of device (Supplementary Fig. S3), the relatively pure white color with (x, y) = (0.31, 0.32) in CIE chart is obtained with the ratio of ZnS:Cu,Mn and ZnS:Cu at 1.8:1.0. Furthermore, the emission intensity can also be dependent on the applied voltage (Fig. 4b). The device initiates light emission at a voltage of around 80 V, after that, the emission intensity increases rapidly. The relation between the relative bias voltages and EL intensity can be well described by the following equation:

1 $${\rm{L}}={{\rm{L}}}_{0}\,\exp \,(\,-\,{\rm{b}}/{{\rm{V}}}^{1/2})$$

where L is the luminance, V is the applied voltage, L_0_ and b are constants depend on the particle size of the phosphor, the concentration of the EL powder in the dielectric, the dielectric constant of the embedding medium and the thickness of the emitting layer^24,25^. After a certain bias voltage, the probability of electrons being accelerated to a given energy to excite the luminescent centers increases dramatically, corresponding to the steeply increase in luminescent intensity^26,27^. Figure 4c shows a dependence of the emission intensity and emission color on the applied frequency. The emission band consists of two Gaussian components centered at 456 nm for blue emission and 511 nm for green emission together with 582 nm from the ZnS:Cu,Mn phosphor. Interestingly, with the increase of applied frequency, there is a shifting ratio between blue emission band and green emission band toward the blue emission band, which is properly due to that green emission of ZnS:Cu phosphor is more intense at low frequencies while blue band predominates at high frequency^28^. Moreover, the emission intensity increase with the applied frequency. Noted that the emission color does not change when the applied voltage increase, suggesting voltage has no influence on spectral distribution of light emission (Fig. 4d).

Moreover, this flexible devices possess an advantage of low power consumption and little heat is generated during long-term operation (Fig. 4e), and its surface temperature does not vary significantly, which is beneficial for their integration with wearable/portable electronics^29^. Meanwhile, the flexibility test is evaluated by the repetitive bending and relaxing of this device (Fig. 4f), no significant degradation of light intensity is observed after the continuous bending curvature from 5 mm to 40 mm. Furthermore, the blue emission devices exhibit similar optoelectronic characteristics compared to the white emission device mentioned above (Supplementary Fig. S4).

**Figure 5:**
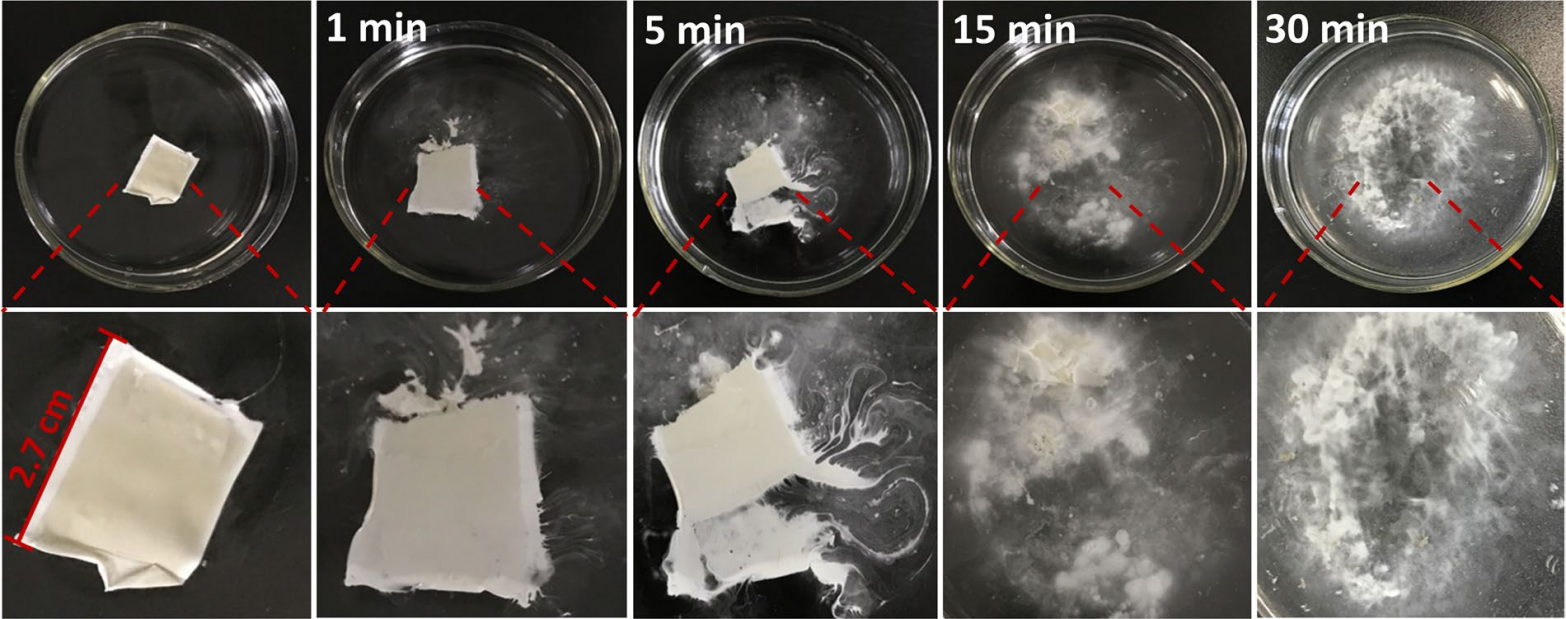
Optical microscope images of the transient ACEL device. The images recording the dissolving of the sandwich structure Ag NWs/phosphor-PVP/AgNWs ACEL device in DI water at room temperature (Upper). Magnified optical images of the reaction of devices with water (Lower).

To evaluate the transient behavior of white ACEL devices, we observe the time-dependent dissolution process of the fabricated device after being immersed in DI water at room temperature (Fig. 5). The edge region of the device start dissolving after being placed in water for 1 min. It has been observed that the whole device is totally dissolved and physically disintegrate in water after 30 min, thus suggesting the capacity to use such water-soluble materials (PVA, PVP, Ag NWs, and phosphor  microparticles) in conjunction with alternating current electroluminescent devices as an effective design approach for transient and flexible light emitting devices. This transient feature of fabricated ACEL devices provide a powerful and highly expedient strategy to solve e-waste and alleviate the environmental pollution problem from abandoned lighting and display electronics.

## Discussion

ZnS:Cu is a widely available phosphor material with well-studied and understood light emission behavior^30^. The emission wavelength from ZnS is determined primarily by the doping activators^26^. In the past decade, many efforts has been devoted to the functional construction of phosphor-based alternating current electroluminescent devices for flexible and stretchable light emission devices, owing to their simple fabrication procedure, low power consumption, large-area production as well as potential cost-effectiveness^31,32^. However, these EL devices exhibit relatively low luminance compared with organic light emitting devices^33^. In the following studies, we will focus on the improvement of the luminance of the transient light emitting devices. Due to frequency- dependent emission intensity property of our devices, luminance enhancement could be expected once a high frequency voltage is applied. Otherwise, low dimensional nanostructures with unique structure and electrical properties, such as carbon nanotube^34^, metal nanowires^35^, and graphene^36^, have been used and investigated to enhance the local electrical field, which improves the probability of electron injection to the active center of phosphor at a relatively low operating voltage. Moreover, some special materials with high dielectric permittivity in the active layer, such as BaTiO_3_ particles, have been well reported to realize high brightness^37^.

In conclusion, transient ACEL devices with a sandwich configuration of Ag NWs/Phosphor-Polymer/Ag NWs have been fabricated through full solution process. Taking advantages of flexible transparent Ag NW networks as both top and bottom electrodes, the fabricated devices exhibit a desirable high flexibility, scalable architecture and both ends side light emission. Moreover, white and blue light emission have been achieved by adjusting the mass ratio of ZnS:Cu,Mn and ZnS:Cu phosphors in active medium layer. The emission color and luminescent intensity are strongly dependent on the applied voltage frequency. Impressively, the large-area flexible ACEL devices demonstrate can be dissolved rapidly in water within 30 min owing to the use of dissolvable Ag NW electrodes and phosphor emissions. The concept reported here establish a baseline of materials choices, device designs, and fabrication approaches for transient light emitting system. Combining such advances in transient electronics with simple structure, common materials, full solution process, and high solubility will demonstrate great potential in resolving the e-waste from abandoned light-emitting products and expand the opportunities for air-stable flexible light emitting devices.

## Methods

### Chemicals

Polyvinyl alcohol (1788, MW = 44.05) and polyvinyl pyrrolidon (MW = 1300000) were purchased from Aladdin Chemicals. Ag NWs were synthesized according to the reported work^38^. ZnS:Cu and ZnS:Cu,Mn phosphor powders were purchased from Shanghai KPT Co. Ethanol, polydimethylsiloxane (PDMS) was supplied from Aladdin Chemicals. Water (18.2 MΩ·cm) was purified using a Milli-Q purification system (DZG-303A).

### Preparation of AgNW-PVA electrodes

The Ag NWs were dispersed in ethanol and then were deposited onto a pre-cleaned plastic petri dishes substrate by spray coating on a 50 °C hot plate with a rectangle shadow mask. The pure PVA aqueous solution with a concentration of 30 mg/ml was poured into plastic petri dishes and cured at 60 °C for 5 h. Next, the AgNW-PVA electrode was peeled off from the plastic substrate and transferred onto the PDMS surface for further fabrication.

### Fabrication of transient ACEL device

10 ml mixed solution of ethanol (as the solvent), ZnS:Cu (1.43 g), ZnS:Cu,Mn (2.57 g) and PVP (2 g) were prepared as white light emitting material. Next, the mixed solution was spun coat onto the AgNW-PVA electrode at a spin rate of 800 rpm for 60 s and cured at 50 °C for 2 h. Then, the Ag NWs and PVP solution (30 mg/ml) were sequentially spray-coated onto the emission layer on a 50 °C hot plate with a rectangle shadow mask. Finally, the whole fabricated device was peeled off the PDMS substrate for further characterization.

### Characterization

SEM images of the samples were taken using a field-emission SEM (JSM-7800F). The cycling bending-unbending tests were performed on a motorized linear stage with built-in controller (WDM-500). The sheet resistance were measured using a standard four-point probe technology (ST2253 purchased from Suzhou Jingge Electronic Co.). Transmittance spectra were recorded using a Shimadzu UV-1750 spectrophotometer. The temperature-time spectra was obtained by the infrared thermometer. The device was powered by AC power (AN97000H purchased from Ainuo Instrument Co., Ltd) and the luminance of the device was measured by a spectrophotometer PR745 (6745-1001-00) and Keithley 2400. All electrical measurements were carried out in an ambient air environment.

## Electronic supplementary material

Supplementary Information
