# Supplementary material for: Transient Light Emitting Devices Based on Soluble Polymer Composites
Source: Sci Rep. 2018 Apr 23;8:6408. doi: 10.1038/s41598-018-24816-y (PMC5913138; doi:10.1038/s41598-018-24816-y)
Supplement: Supplementary file 1 — Supplementary Information [file 41598_2018_24816_MOESM1_ESM.doc]

**Supplementary Information**

**Transient Light Emitting Devices Based on Soluble Polymer Composites**

Yingying Chen^1^, Hang Lu^1^, Fei Xiu^1^, Tao Sun^1^, Yamei Ding^1^, Juqing Liu^1^ & Wei Huang^1, 2^

^1^ Key Laboratory of Flexible Electronics (KLOFE) & Institute of Advanced Materials (IAM), Jiangsu National Synergetic Innovation Center for Advanced Materials (SICAM), Nanjing Tech University (NanjingTech), 30 South Puzhu Road, Nanjing 211816, China.

^2^ Key Laboratory for Organic Electronics and Information Displays & Institute of Advanced Materials (IAM), SICAM, Nanjing University of Posts & Telecommunications, 9 Wenyuan Road, Nanjing 210023, China.

Correspondence and requests for materials should be addressed to J.Q.L (email:

[iamjqliu@njtech.edu.cn](mailto:iamjqliu@njtech.edu.cn)) or to W.H. (email: iam[whuang@njtech.edu.cn](mailto:whuang@njtech.edu.cn))

**Supplementary Figure 1. The characteristics of AgNW-PVA electrode.** a) Relative resistance change of the AgNW-PVA electrode during repeated flat-bending test. b) Transmittance spectra of AgNW-PVA electrode with four different sheet resistances.

**Supplementary Figure 2. Photographs of the large-area flexible device under flat and mechanical bending state with different size and shape patterns.**

**Supplementary Figure 3.** CIE coordinates of the device corresponding to five different phosphors proportions.

**Supplementary Figure** **4. Optoelectronic characterization of pure blue phosphors-based device.** (a) Luminance versus alternating voltage properties of the device under different frequencies. (b) CIE coordinates of (0.171, 0.249) for the device. (c) The normalized EL spectra. Relative EL emission spectra at different (d) voltages and (e) frequencies. (f) Photograph and schematic illustration of blue ACEL device as a function of the bending radius.
